# Breast Tissue 3D Segmentation and Visualization on MRI

**DOI:** 10.1155/2013/859746

**Published:** 2013-07-29

**Authors:** Hong Song, Xiangfei Cui, Feifei Sun

**Affiliations:** ^1^School of Software, Beijing Institute of Technology, 5 South Zhongguancun Street, Haidian District, Beijing 100081, China; ^2^School of Computer Science & Technology, Beijing Institute of Technology, 5 South Zhongguancun Street, Haidian District, Beijing 100081, China

## Abstract

Tissue segmentation and visualization are useful for breast lesion detection and quantitative analysis. In this paper, a 3D segmentation algorithm based on Kernel-based Fuzzy C-Means (KFCM) is proposed to separate the breast MR images into different tissues. Then, an improved volume rendering algorithm based on a new transfer function model is applied to implement 3D breast visualization. Experimental results have been shown visually and have achieved reasonable consistency.

## 1. Introduction

Recently, magnetic resonance imaging (MRI) technique has been widely used in diagnosing and detecting diseases. It provides an effective mean of noninvasively mapping the anatomy of a subject. It works better than X-ray computed tomography (CT) at soft tissue, such as breast. The three-dimensional segmentation and visualization of breast are useful for breast lesion detection and quantitative analysis.

Segmentation is applied to extract the interesting tissues in the breast. Several algorithms have been developed for segmenting the breast tissues. Threshold-based method, the gradient method, polynomial approximation method, the active contour models, and classifier segmentation are used in breast skin segmentation. Raba et al. [1] summarized that threshold-based method, the gradient method, polynomial approximation method, the active contour models, and classifier segmentation are the main methods commonly used in breast skin segmentation. Chen et al. [2] introduced the fuzzy clustering algorithm to the tumor region segmentation which had achieved better results. Kannan et al. [3] made the breast region segmentation by introducing new objective function of fuzzy c-means with the help of hypertangent function, Lagrangian multipliers method, and kernel functions. However, these studies did not separate the fat and fibroglandular tissues. Pathmanathan [4] suggested a region-growing method, which required the user to manually choose one or more seed points. This method got satisfying results, but it is inefficient and time consuming. Nie [5] used two steps to segment the breast: firstly, locating the skin border and lungs region by standard FCM algorithm and secondly, extracting the fibroglandular tissue by an adaptive FCM algorithm. However, it is a semiautomated method.

Two kinds of methods are mainly applied in volume visualization, which are surface rendering and volume rendering. For surface rendering, Marching Cubes (MC) algorithm [6] is usually used which was developed by Lorensen and Cline in 1987. MC represents 3D objects by surface representations such as triangular patches or polygonal meshes. However, MC algorithm suffered from a common problem of having to make a binary classification: either a surface passes through the current voxel or it does not [7]. Volume rendering addresses these defects, which can display the dataset by translucent images. By volume rendering, the internal structure can be seen and analyzed conveniently, which is useful for breast disease diagnosis. Most of the volume rendering algorithms are based on the optical theory [8]. The optical model can simulate the propagation of light in real world. Levoy [7] proposed ray-casting algorithm to display surfaces from volume data. Lacroute [9] improved ray casting with shear-warp algorithm. Maximum intensity projection (MIP) is a variant of volume rendering in which the color of the pixel in the final image is determined by the maximum value encountered along a ray.

**Figure 1:**
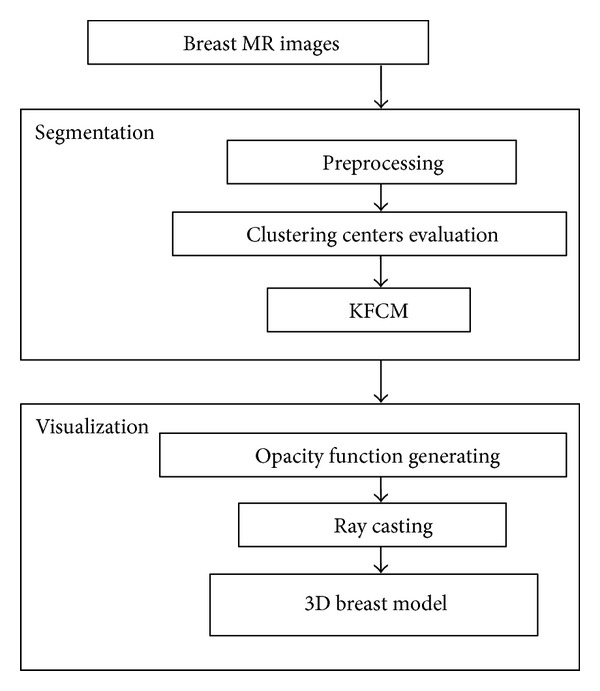
Overview of breast segmentation and visualization.

In this paper, firstly, an automatic segmentation algorithm based on KFCM is developed to separate breast images into different tissues. Secondly, an improved volume rendering algorithm based on a new transfer function model is proposed to visualize the breast on MRI.  Figure 1 shows the procedure of the breast segmentation and visualization.

## 2. Breast Tissue Segmentation

Breast tissue segmentation is used to extract the interesting regions. It separates the breast into three parts: fat tissue, fibroglandular tissue, and air. In this paper, an automatic segmentation algorithm is developed, which consists of three steps described as follows.

### 2.1. Image Preprocessing

**Figure 2:**
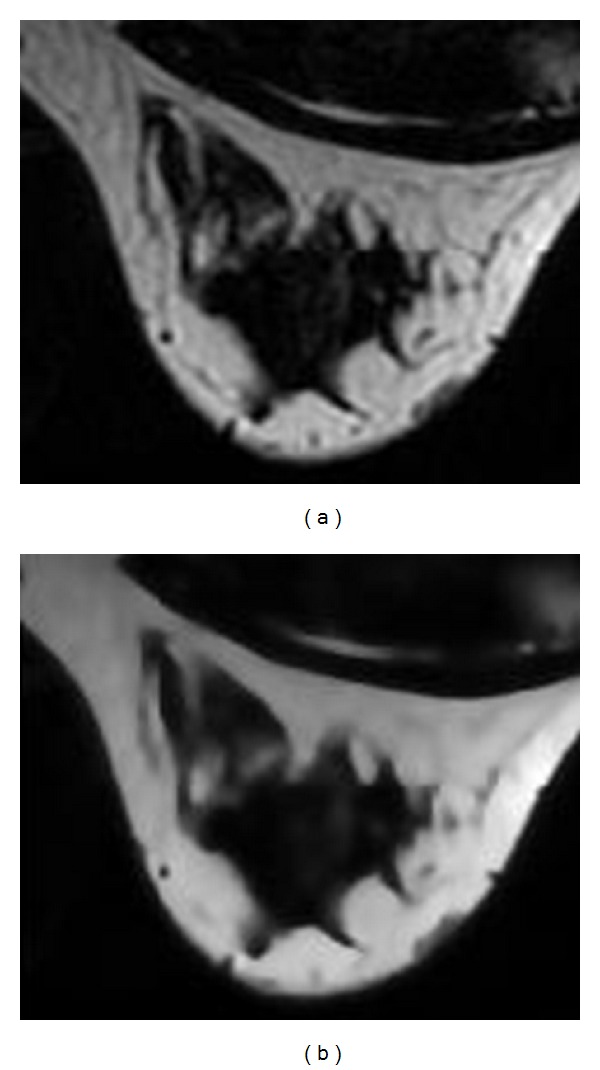
Breast image preprocessing ((a) original image; (b) after preprocessing).

MR image has inhomogeneity, noise, and other factors which affect the continuity and accuracy of the images segmentation results. Therefore, the anisotropic diffusion filter [10] is introduced to reduce the image noise. And then binarization is performed on the image by using Otsu's thresholding algorithm. Since air produces almost zero MR signals, the background in MR images is virtually black, and the breast-skin boundary is relatively clear. Pectoral muscle appears dark gray in the axial T1-weighted breast images, and the grey level of this area is close to zero. So the direct threshold-based method is used to segment the breast region.  Figure 2(a) is the original MR image that is partially enlarged.  Figure 2(b) is the image that is processed by anisotropic diffusion filter which is smoother than the original image while the edges are well preserved.

### 2.2. Kernel-Based Fuzzy C-Means Clustering Algorithm

Typical Fuzzy C-Means (FCM) clustering algorithm is improved from C-means method, in which every iterative makes the sample belong to an exact cluster [11]. 

FCM introduces a fuzzy membership function which controls the degree of a sample belonging to different classes. The Kernel-based Fuzzy C-Means (KFCM) clustering method was proposed by Zhang [12, 13] based on FCM. It used a kernel function Φ(*x*) instead of the original Euclidian norm metric in typical FCM algorithm. The KFCM algorithm minimized the following objective function:

(1) $$\begin{array}{c} J_{kfcm}=\sum_{j=1}^{N}\;\sum_{i=1}^{c}p_{ij}^{m}{||\Phi \left(x_{j}\right)-\Phi \left(v_{i}\right)||}^{2}, \end{array}$$

where *p*_*ij*_^*m*^ is a fuzzy membership matrix. *c* is the number of clustering centers which is usually set by a priori knowledge. *N* is the number of sample points. *v*_*i*_ is the fuzzy center of the *i*th cluster. The parameter *m* is a constant which controls the fuzziness of the resulting partition. In the experiments, *m* is set as 2.

In this paper, the Gaussian function (see (2)) is selected as the kernel function:

(2) $$\begin{array}{c} K\left(x,y\right)=e^{-{||x-y||}^{2}/{\left(2\sigma \right)}^{2}}. \end{array}$$

So the distance measure and the objective function can be redefined as

(3) ||Φ(xj)−Φ(vi)||2=K(xj,xk)+K(vi,vi)−2K(xj,vi)=2−2K(xj,vi),Jkfcm=∑j=1N ∑i=1c2pijm(1−K(xj,vi)).

The equations of clustering center and membership matrix are similar with FCM as follows:

(4) $$\begin{array}{c} p_{ij}=\frac{{\left(1-k(x_{j}-v_{i})\right)}^{1/(1-m)}}{\sum_{i=1}^{c}{\left(1-k(x_{j},v_{i})\right)}^{1/(1-m)}},\\ v_{i}=\frac{\sum_{k=1}^{N}p_{ik}^{m}k\left(x_{k},v_{i}\right)x_{k}}{\sum_{k=1}^{N}p_{ik}^{m}k\left(x_{j},v_{i}\right)}. \end{array}$$

Because the sum of every column value in the fuzzy membership matrix *p*_*ij*_^*m*^ is 1,

(5) $$\begin{array}{c} \sum_{i=1}^{c}p_{ij}=1\;\left(j=1,2,\ldots ,N\right), \end{array}$$

the result of KFCM is influenced by the number of the clustering centers. The result will be devious if the number is very different from the reality. However, the classification for breast using KFCM benefits from the good performance of convergence [11] because we know exactly the clustering number.

### 2.3. Evaluation of the Clustering Centers

The clustering centers of KFCM are initialized randomly without analyzing the original dataset, which probably causes nonaccurate segmentation and needs more iteration times. So we utilize a boundary model to determine the rough boundary of breast tissues and then calculate more accurate clustering centers.

We assume that there are gradual changes in data value between different tissues. For scalar data, the gradient is a first derivative measure which describes the direction of greatest change. The gradient magnitude is a scalar quantity that represents the rate of change in the scalar field. Since precise boundary is unnecessary, the second directional derivative is abstained for reducing complexity. *f*′ is used to represent the gradient magnitude, where *f* is the scalar function:

(6) $$\begin{array}{c} f^{\prime }=||\nabla f||. \end{array}$$

We assume that if we order the tissues by data value, then each type touches only types adjacent to it in the ordering [7]. The transition from one tissue to another is smooth. We create a 1D histogram of *f*′ and find its peaks. The positions where peaks appear are approximately the boundary. We denote the boundary by

(7) $$\begin{array}{c} \Psi \left(x\right)\;x=1,2,\ldots ,N. \end{array}$$

*N* is the number of positions whose gradient magnitude belongs to the peaks. There are three kinds of tissues in breast MR images (fat, fibroglandular, and air). So Ψ_1_(*x*), Ψ_2_(*x*), and Ψ_3_(*x*) will be produced. And then compute the one moment of the discrete boundary points set [14]:

(8) $$\begin{array}{c} m_{pq}=\sum_{j=1}^{N}\;\sum_{i=1}^{N}i^{p}j^{q}f\left(i,j\right),\\ \mu_{pq}=\sum_{j=1}^{N}\;\sum_{i=1}^{N}{\left(i-i_{c}\right)}^{p}{\left(j-j_{c}\right)}^{q}f\left(i,j\right), \end{array}$$

where *i*_*c*_ = *m*_10_/*m*_00_,  *j*_*c*_ = *m*_01_/*m*_00_.

So the one moment can be used to initialize the clustering centers to perform the KFCM algorithm. 

## 3. Breast Visualization

Volume rendering is useful for exploring the internal structure of the object such as breast. Most of the volume rendering algorithms are based on the optical theory. There are several different optical models for light interaction with volume densities of absorbing, emitting, reflecting, and scattering materials [8]. In this paper only absorption plus emission is considered in which voxel emits light itself and absorbs incoming light. It is the most common one in volume rendering [15]. Each pixel of the image casts a single ray into the dataset. The ray interacts with the scalar value of the dataset which has been virtually mapped to color and optical properties. The mapping is implemented through a transfer function. The optical properties then are used in compositing procedure which is known as the volume rendering integral. The integral is solved numerically to get the color of the pixel at last. This process continues until the color of all the pixels of the image is obtained, and then the final image will be displayed.

### 3.1. Optical Model and Composition

Every pixel casts a ray from viewing image to the volume, and then we resample the volume scalar data values at equispaced intervals through trilinear interpolation. The optical model and composition are not the main point of this paper and are presented here for completeness.

The process of the light propagation and the composition is parameterized. A ray cast into the volume is represented by *x*(*t*), where *t* is the distance from the eye to the current position. The scalar value along *x*(*t*) is denoted by *s*(*x*(*t*)). Since only absorption and emission are considered, the volume rendering equation integrates absorption coefficients and emissive colors:

(9) $$\begin{array}{c} c\left(t\right)=c\left(s\left(x\left(t\right)\right)\right),\\ k\left(t\right)=k\left(s\left(x\left(t\right)\right)\right). \end{array}$$

Both *c*(*t*) and *k*(*t*) are functions of distance *t* instead of scalar *s*. We denote the energy which eventually reaches the eye from *t* = *d* by *c*′. If *k*(*t*) is constant along the ray,

(10) $$\begin{array}{c} c^{\prime }=c\cdot e^{-kd\;}. \end{array}$$

However, *k*(*t*) is usually not constant. It depends on the distance from the eye, so

(11) $$\begin{array}{c} c^{\prime }=c\cdot e^{-\int_{0}^{d}k(t)dt}. \end{array}$$

So, the integral over the absorption coefficients in the exponent is

(12) $$\begin{array}{c} T\left(d_{1},d_{2}\right)=\int_{d_{2}}^{d_{1}}k\left(t\right)dt, \end{array}$$

which is also called the optical depth [15]. It is a single point. We should perform integral for all possible positions *t* along the ray to acquire the total amount of radiant energy *C* reaching the eye from direction:

(13) $$\begin{array}{c} C=\int_{0}^{\infty }c\left(t\right)\cdot e^{-T(0,t)dt}. \end{array}$$

The integral of (12) can be approximated by a Riemann sum:

(14) $$\begin{array}{c} T\left(0,t\right)\approx \sum_{i=0}^{\left\lfloor t/\Delta t\right\rfloor }k\left(i\cdot \Delta t\right)\Delta t, \end{array}$$

where Δ*t* represents the distance between resampling points.

According to exponential formula, the component of (13) can be redefined as

(15) $$\begin{array}{c} e^{-T(0,t)}=\prod_{i=0}^{\left\lfloor t/\Delta t\right\rfloor }e^{-k(i\cdot \Delta t)\Delta t}. \end{array}$$

The approximate evaluation of the volume rendering integral is

(16) $$\begin{array}{c} C=\sum_{i=0}^{n}C_{i}\prod_{j=0}^{i-1}\left(1-A_{j}\right), \end{array}$$

where *n* is the number of samples, *A*_*j*_ is called opacity, and *C*_*i*_ is the emitted color of the *i*th ray segment:

(17) $$\begin{array}{c} A_{i}=1-e^{-k(i*\Delta t)\Delta t}, \end{array}$$

(18) $$\begin{array}{c} C_{i}=c\left(i\cdot \Delta t\right)\Delta t. \end{array}$$

In our case, the front-to-back order is applied by stepping *i* from 1 to *n*. The following iterative addresses (16):

(19) $$\begin{array}{c} C_{i}^{\prime }=C_{i+1}^{\prime }+\left(1-A_{i-1}^{\prime }\right)C_{i},\\ A_{i}^{\prime }=A_{i-1}^{\prime }+\left(1-A_{i-1}^{\prime }\right)A_{i} \end{array}$$

So we can get one pixel of the final image. After all the pixels of the final image are integrated from all directions, the result of volume rendering will be displayed.

### 3.2. A New Transfer Function Model

**Figure 3:**
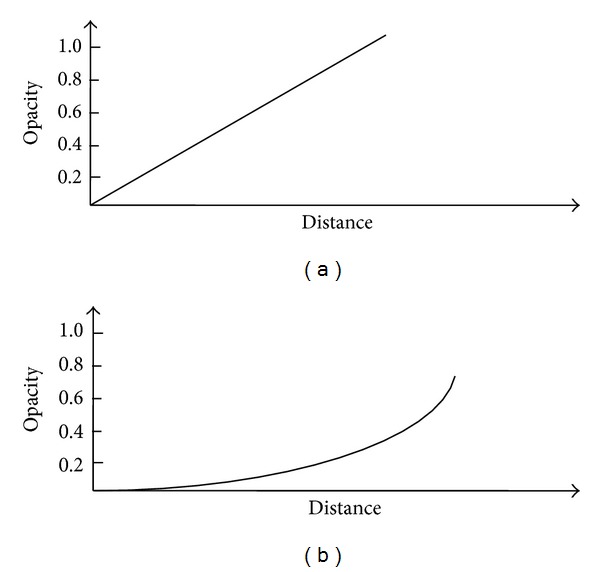
(a) Linear gradient. (b) Quadratic gradient.

Function *a*(*x*) which is termed the opacity distribution function is introduced [16], which maps data value in an identical tissue to opacity. The independent variable is the Euclidean distance between data values and clustering centers, which are generated through KFCM. This function can be a piecewise function, polynomial function, or spline. In Figure 3, two opacity distribution functions are demonstrated. In Figure 3(a), the opacity has a linear gradient from the center of the sphere to the outline. In Figure 3(b), the opacity has a quadratic gradient in which we can largely concentrate on the boundary of the material.

### 3.3. Performance Optimization

**Figure 4:**
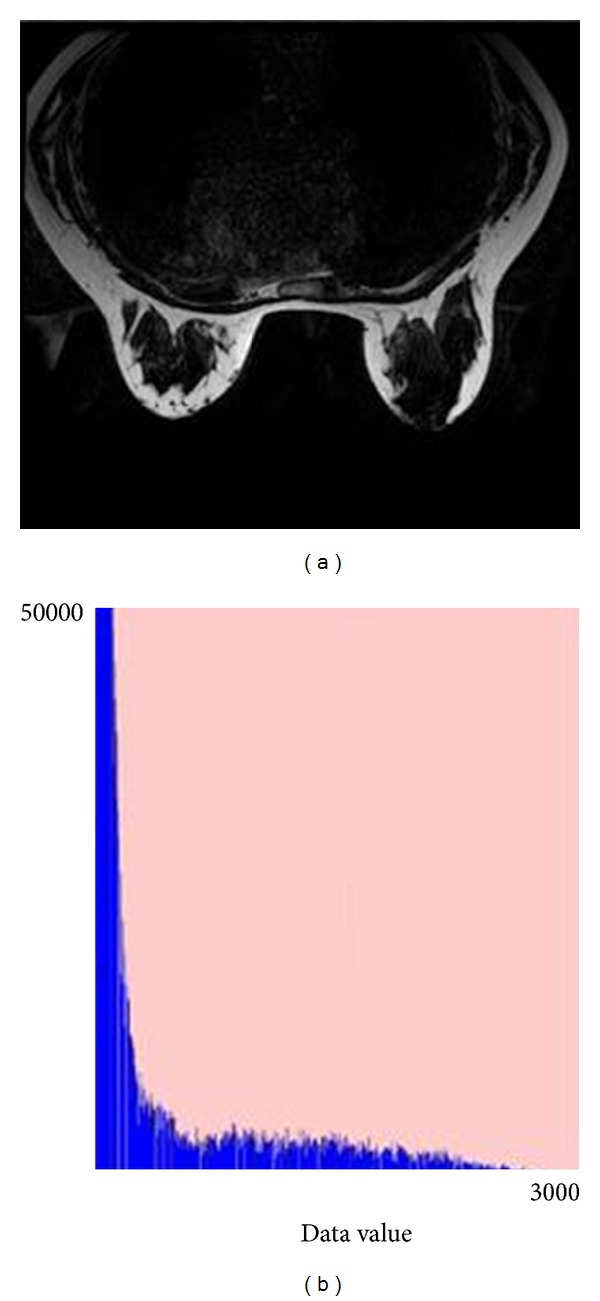
Breast MR image and its histogram. ((a) original MRI; (b) histogram).

**Figure 5:**
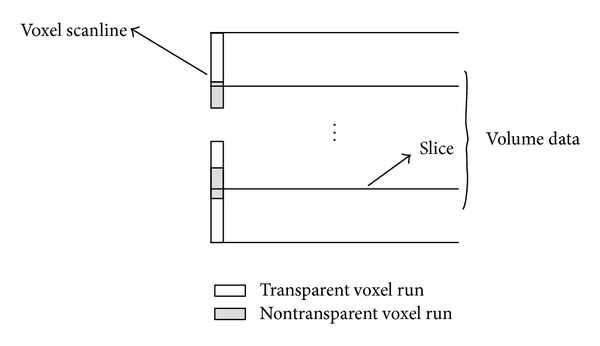
Scanline is perpendicular to the slices and skips the transparent region.

According to Figure 4, the histogram of data values of one slice in the dataset implies that the data value of many points is zero. They should deserve little attention. But in traditional ray-cast algorithm, we have to consider the voxels which contribute nothing to the final image. Therefore, a scanline that is perpendicular to the slices is introduced (Figure 5). The scanline consists of gray segments and white segments. The white segments represent transparent parts in the volume, which contribute little to the final image. So, the interpolation in preprocess and the iterative of (19) are abstained in transparent segments of the corresponding scanline. Therefore, the compositing speeds up.

## 4. Results and Discussion

The breast MR images we used in this paper were acquired from the Military General Hospital of Beijing PLA. There are 28 slices of 512 × 512 samples each. The procedure was implemented on a 3.4 GHz CPU, 2 G memory PC. 

**Figure 6:**
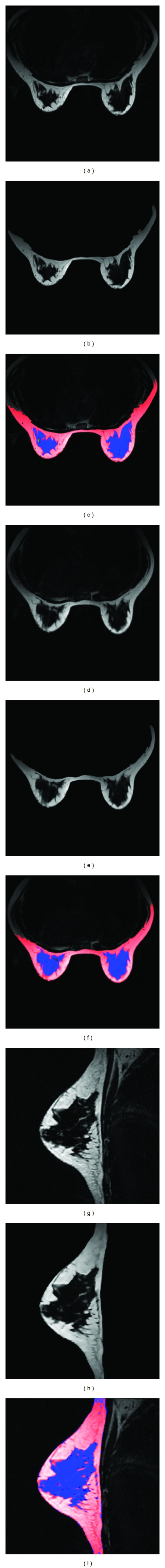
The results of segmentation in 2D field. ((a),(d), and (g) original images; (b), (e), and (h) the results of preprocessing; (c), (f), and (i) the result of KFCM).

Figure 6 shows the results of KFCM algorithm of three slices in the dataset. This example indicates the effectiveness of KFCM algorithm in 2D field. Fibroglandular and fat tissues can be relatively separated precisely. The blue region is fibroglandular tissue. The shortcoming of KFCM is that the convergence time cannot be predicted and may be unacceptably long. However, through the initialization of clustering centers in Section 2.3, the convergence time can be made more acceptable.

**Figure 7:**
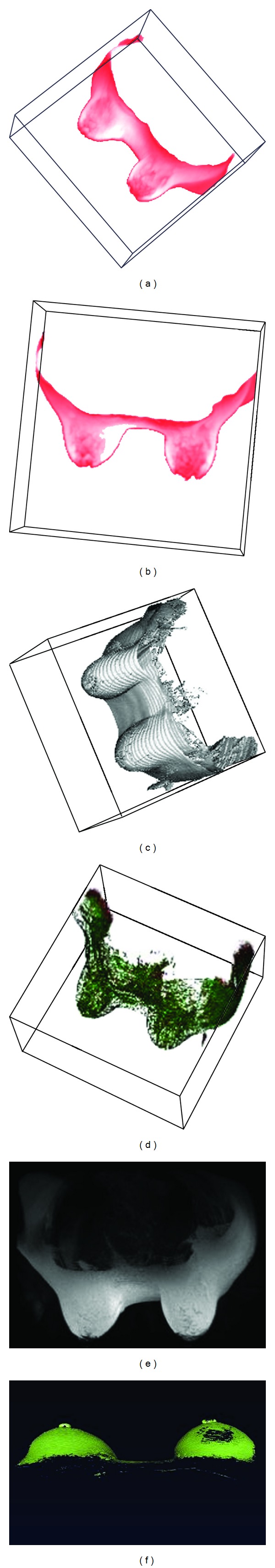
Results of 3D segmentation and visualization. ((a), (b) Results of segmentation and visualization based on proposed algorithms; (c) result of Marching Cubes; (d) result of ray casting with a linear transfer function; (e) result of MIP; (f) result of ImageVis3D).

Figure 7 shows the results of the breast visualization through various algorithms. In Figure 7(c), we can see that the result of Marching Cubes algorithm can well display the breast skin, but the internal structure cannot be seen. A simple linear transfer function was used to produce a result with misty fibroglandular tissue, as shown in Figure 7(d). The green regions represent fibroglandular tissue, and the white regions represent fat tissue. They are mixed together, so we could not easily distinguish them. 

Figure 7(e) shows the result of MIP. MIP uses the maximum value encountered along a ray to determine the color, so it is difficult to design the transfer function. However, the region having the maximum value may not be the interested region, and furthermore it does not consider the contribution of the other points along the ray.

Figure 7(f) shows the results of ImageVis3D system which is developed by the Center for Integrative Biomedical Computing, University of Utah. This system can visualize the biological tissue well, but the transfer function should be adjusted manually, and the quality of the rendered image depends on the user's experience.

Figures 7(a) and 7(b) show the visualization of breast by KFCM algorithm. The number of clustering centers in KFCM is three: fat tissue, fibroglandular tissue, and air. The comparison of convergence time is shown in Table 1. We have tested a single dataset for three times. From Table 1, we can see that the convergence time is stably six minutes with the evaluation of the clustering center proposed in Section 2.3. It is faster and more predicable than KFCM with random clustering centers. Then, using the compositing algorithm described in Section 3.1 and the quadratic opacity distribution function described in Section 3.2, two views are computed. Comparing with the other results, Figures 7(a) and 7(b) show the fibroglandular more clearly. Fibroglandular tissue is separated from fat tissue well. The pink regions in the breast represent fibroglandular tissue, and the white regions in the breast represent fat tissue.

## 5. Conclusion

A 3D segmentation algorithm based on Kernel-based Fuzzy C-Means (KFCM) is presented to separate the breast images into different tissues. In the KFCM algorithm, we propose to evaluate the clustering centers through the rough boundary of breast tissues before clustering process. Then, an improved volume rendering algorithm based on a new transfer function model is applied to implement 3D breast visualization. Experimental results show that our algorithm can efficiently segment fibroglandular and fat tissues. Also, the visualization performance of our algorithm is better than ray-casting algorithm with a linear transfer function and MIP, which makes it useful for breast lesion detection and quantitative analysis.

## Figures and Tables

**Table 1:** KFCM convergence time.

Test order	KFCM with evaluation of clustering center (mins)	KFCM with random clustering center (mins)
1	6	21
2	6	15
3	6	32
